# Crystal structure and Hirshfeld surface analysis of [FeCl_4_(*L*H)_2_] (*L*H = 1*H*-imidazo[4,5-*b*]pyridin-4-ium)

**DOI:** 10.1107/S2056989025010564

**Published:** 2026-01-01

**Authors:** Soffa Imene, Bouhidel Zakaria, Sahli Kaouther, Cherouana Aouatef, Bendeif El-Eulmi

**Affiliations:** a Unité de Recherche de Chimie de l'Environnement et Moléculaire Structurale (URCHEMS) Département de Chimie Université Mentouri de Constantine 25000 Constantine Algeria; b Pharmaceutical Sciences Research Center CRSP, Constantine 25000, Algeria; c Synchrotron SOLEIL, L’Orme des Merisiers, BP48, Saint Aubin, 91192, Gif-sur-Yvette, France; d Laboratoire de Cristallographie, Résonance Magnétique et Modélisation CRM2 UMR 7036 Institut Jean Barriol Faculté des Sciences et Technologies BP 70239 54506 Vandoeuvre lès Nancy France; University of Buenos Aires, Argentina

**Keywords:** 4-aza­benzimidazole, iron, crystal structure, Hirshfeld surface analysis

## Abstract

The title coordination complex tetra­chlorido­bis­(1*H*-imidazo[4,5-*b*]pyridin-4-ium-κ*N*^3^)iron(II), [FeCl_4_(C_6_H_6_N_3_)_2_] or [FeCl_4_(LH)_2_], was synthesized and structurally characterized by single-crystal X-ray diffraction. The complex crystallizes in the triclinic space group *P*1. The iron atom (site symmetry ) is hexa-coordinated, adopting a slightly distorted octa­hedral geometry defined by two 1*H*-imidazo[4,5-*b*]pyridinium ligands and four chloride anions. In the crystal, N—H⋯Cl hydrogen bonds generate two-dimensional layers parallel to the *ab* plane, while the three-dimensional supra­molecular framework is further consolidated by C—H⋯Cl inter­actions. In addition, π–π stacking inter­actions contribute to the overall cohesion of the crystal structure. Hirshfeld surface analysis indicates the significance of various inter­molecular contacts in the crystal packing, with major contributions from Cl⋯H/H⋯Cl (43.2%), H⋯H (22.5%), C⋯H/H⋯C (16.4%), H⋯N/N⋯H (4.4%), N⋯C/C⋯N (3.7%), C⋯C (3.6%), Cl⋯N/N⋯Cl (3.2%), Cl⋯C/C⋯Cl (2.4%), and N⋯N (0.6%) inter­actions.

## Abstract 2

The crystal structure of the complex tetra­chlorido­bis­(1*H*-imidazo[4,5-*b*]pyridin-4-ium-κ*N*^3^)iron(II) has been determined. The compound crystallizes in the monoclinic system and shows an octa­hedral coordination environment around the Fe centre.

## 1. Chemical context

The coordination chemistry of iron has attracted great inter­est due to the importance of iron-based coordination compounds and their applications in various fields such as catalysis (Sheldon & Kochi, 1981 ▸; Meunier *et al.*, 2000 ▸), magnetism (Toftlund, 1989 ▸; Gütlich, 1981 ▸) and bioinorganic chemistry, particularly in simulating the behaviour of enzymes involved in electron transfer and oxygen transfer processes (Reedijk & Bouwman, 1999 ▸). Iron is frequently hexa­coordinated in its coordination complexes; it is important to note that the number, type, and geometric arrangement of ligands around the metal centre significantly influence the previously mentioned properties (Börzel *et al.*, 2002 ▸).

Nitro­gen-containing heterocycles, which feature one or more nitro­gen atoms within their ring structures, represent important and distinctive categories of heterocycles (Li *et al.*, 2023 ▸). These heterocycles play an important role in coordination chemistry as N-donor ligands. Among them, benzimidazole is particularly well known and widely used as a nitro­gen-donor ligand (Ana, 2019 ▸).

Benzimidazole contains two N-donor atoms that can coordinate transition metals (Sundberg & Martin, 1974 ▸). The imidazole ring consists of two nitro­gen atoms, one of which is pyrrole-like, with its lone pair electrons contributing to the aromatic sextet. The second N atom is pyridine-like, with a non-delocalized lone pair that imparts basic properties (Haga, 2003 ▸). The substitution of a CH group in the benzene ring with a nitro­gen atom, which is necessarily pyridine-like, imparts a basicity similar to that of nitro­gen in the imidazole ring. This modification enhances the ligand’s basic properties by introducing a second pyridine-like nitro­gen atom, thereby increasing its binding affinity to metal ions (Zapata *et al.*, 2008 ▸). Thus, 4-aza­benzimidazole consists of a fused imidazole and pyridine system. The positioning of its basic nitro­gen atoms enables the potential for chelation with metal ions, facilitating the formation of four-membered rings (Korabik *et al.*, 1998 ▸). The three nitro­gen atoms in 4-aza­benzimidazole, each with distinct characteristics, provide versatility to the ligand, allowing it to coordinate with metal ions in various modes, including monodentate, bidentate, chelating, and bridging arrangements.
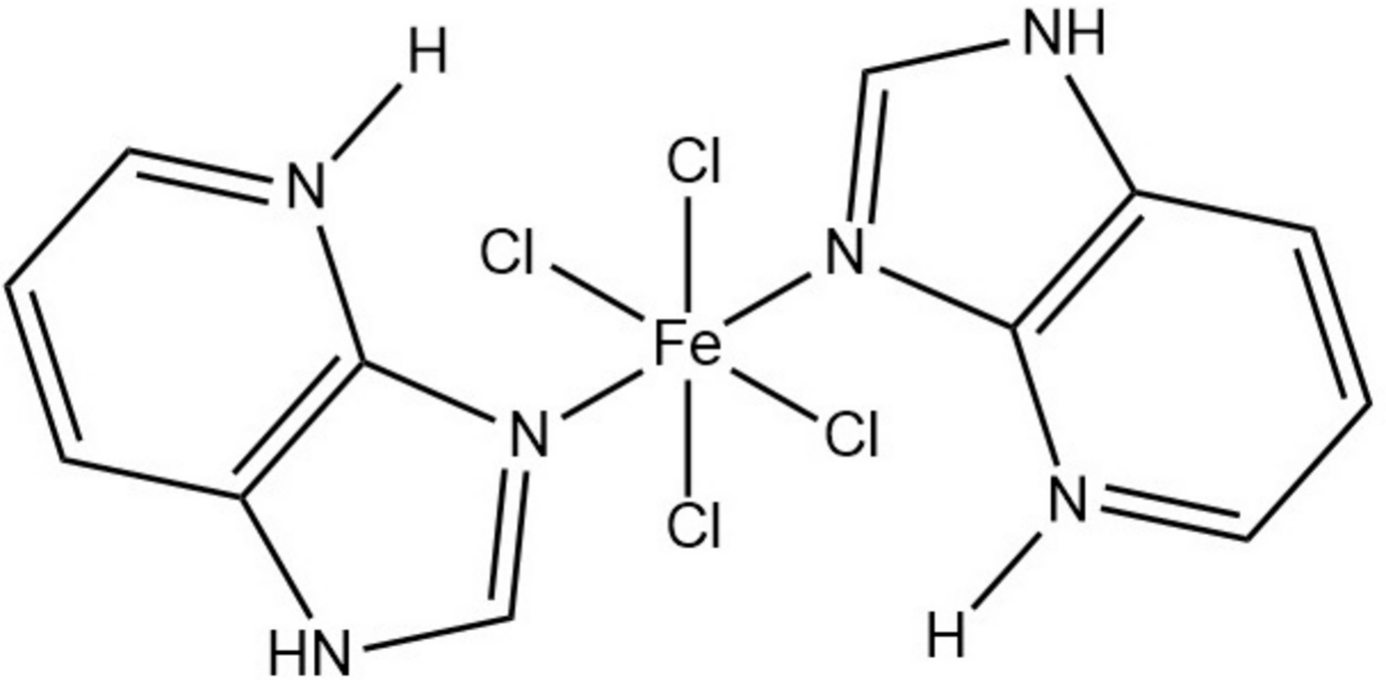


## 2. Structural commentary

**Figure 1:**
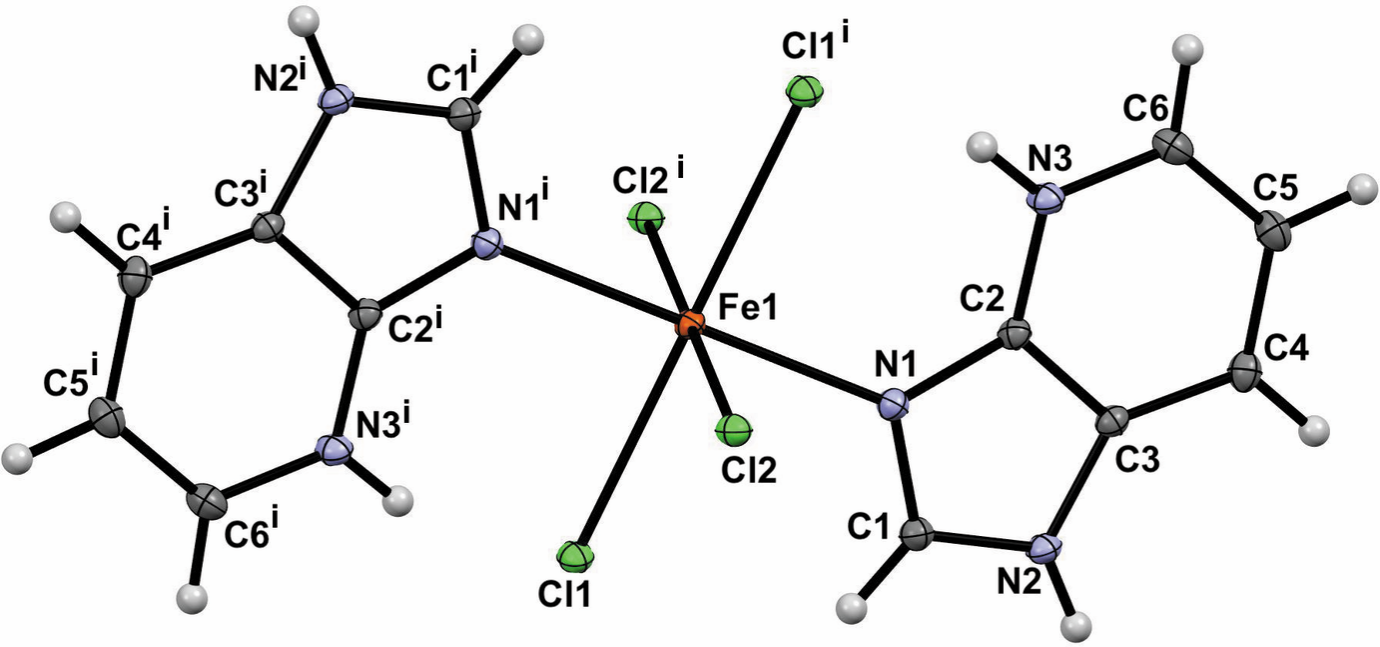
*ORTEP* view of the structure of the title complex showing the atom-labelling scheme. Displacement ellipsoids are drawn at the 50% probability level. Symmetry code: (i) −*x*, −*y*, −*z*.

The title complex crystallizes with the Fe^2+^ atom located on a crystallographic centre of symmetry, generating the complete mol­ecule by inversion symmetry. As a result, the structure consists of two symmetry-related halves forming a discrete mononuclear complex with an octa­hedral geometry and a trans configuration for the N atoms. The Fe^2+^ centre is coordinated by two nitro­gen atoms from two N-donor 1*H*-imidazo[4,5-*b*]pyridinium ligands in the axial positions and four chloride ions in the equatorial plane (Fig. 1 ▸), resulting in an N_2_Cl_4_ coordination environment. The bond lengths and angles (Table 1 ▸) are consistent with a slightly distorted octa­hedral geometry. The axial Fe1—N1 bond length is 2.2256 (9) Å, whereas the equatorial Fe1—Cl1 and Fe1—Cl2 bond lengths are 2.5845 (2) and 2.4564 (2) Å, respectively. These values fall within the expected range for Fe^2+^ octa­hedral complexes reported in the literature (Rettig *et al.*, 2000 ▸; Rusbridge *et al.*, 2018 ▸). The bond angles around the metal centre are close to the ideal values of 90°, with only minor deviations, confirming a near-ideal octa­hedral coordination geometry. Moreover, the iron(II) atom lies within the equatorial plane, while the five-membered imidazole and six-membered pyridine rings of the chelating ligand are approximately coplanar, allowing for efficient coordination and contributing to the overall planarity of the ligand framework. The cohesion of the mol­ecular structure is achieved through an intra­molecular N—H⋯Cl hydrogen bond formed between the nitro­gen atom of the imidazole ring and a chloride ion acceptor [N3—H3⋯Cl1^i^; symmetry code: (i) −*x*, −*y*, −*z*).

## 3. Supra­molecular features

**Figure 2:**
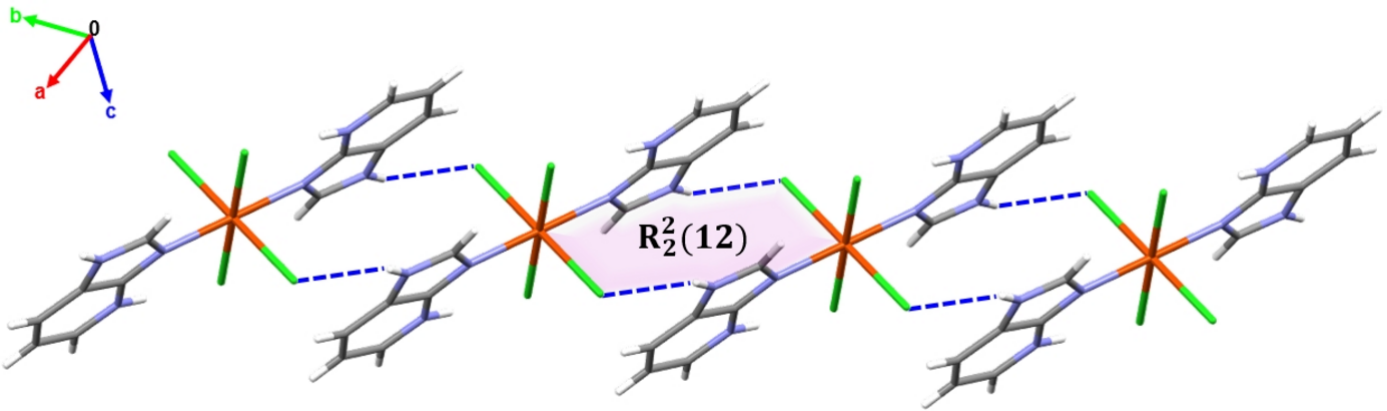
Network propagation along the [110] direction through N2—H2⋯Cl2^ii^ hydrogen bonds, forming *R*^2^_2_(12) graph-set motifs (highlighted in purple).

**Figure 3:**
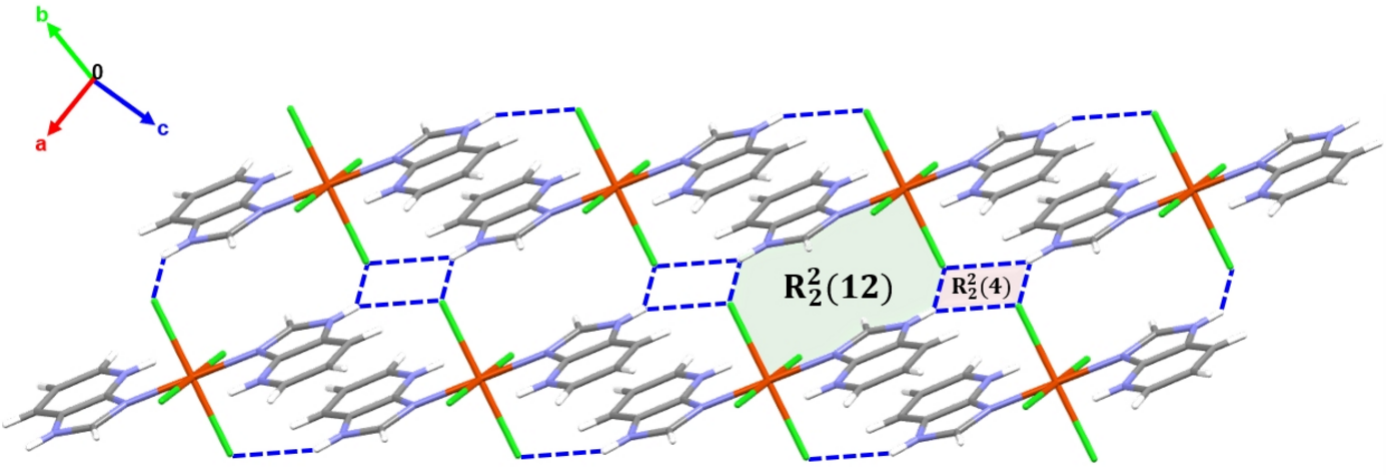
Two-dimensional network of the title compound parallel to the *ab* plane, showing N—H⋯Cl hydrogen bonds and associated ring motifs.

**Figure 4:**
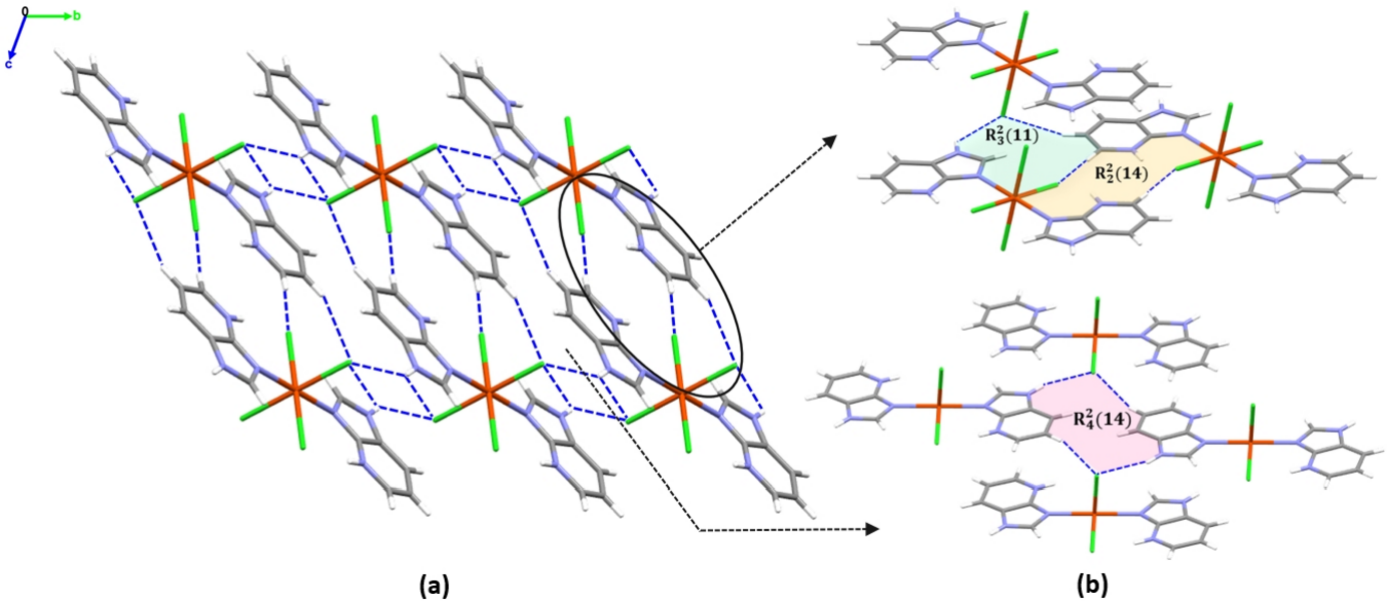
(*a*) Fragment of the supra­molecular crystal structure packing of the title compound, (*b*) hydrogen-bonding patterns. Hydrogen bonds are indicated by dashed blue lines.

The crystal structure of the title compound is consolidated by various supra­molecular inter­actions, including classical and non-classical hydrogen bonds, as well as π–π stacking inter­actions. All N—H and C—H groups of the 1*H*-imidazo[4,5-*b*]pyridinium ligand, except for the C4—H4 group, act as hydrogen-bond donors, while the chloride ions serve as acceptors, forming N—H⋯Cl and C—H⋯Cl inter­actions (Table 2 ▸). Furthermore, N2—H2⋯Cl2^ii^ hydrogen bonding links the mol­ecules into chains along the [110] direction. These chains adopt a ribbon-like arrangement featuring *R*^2^_2_(12) ring motifs (Fig. 2 ▸; Bernstein *et al.*, 1995 ▸). These chains are further connected *via* classical hydrogen bonds (N2—H2⋯Cl2^iii^) into a two-dimensional network lying parallel to the (001) plane, based on alternating *R*^2^_2_(4) and *R*^2^_2_(12) ring motifs (Fig. 3 ▸). Extension into a three-dimensional network occurs *via* non-classical C—H⋯Cl hydrogen bonds C5—H5⋯Cl2^v^ and C6—H6⋯Cl1^iv^, forming *R*^2^_2_(14), *R*^4^_2_(14) and *R*^3^_2_(11) graph-set motifs (Fig. 4 ▸).

**Figure 5:**
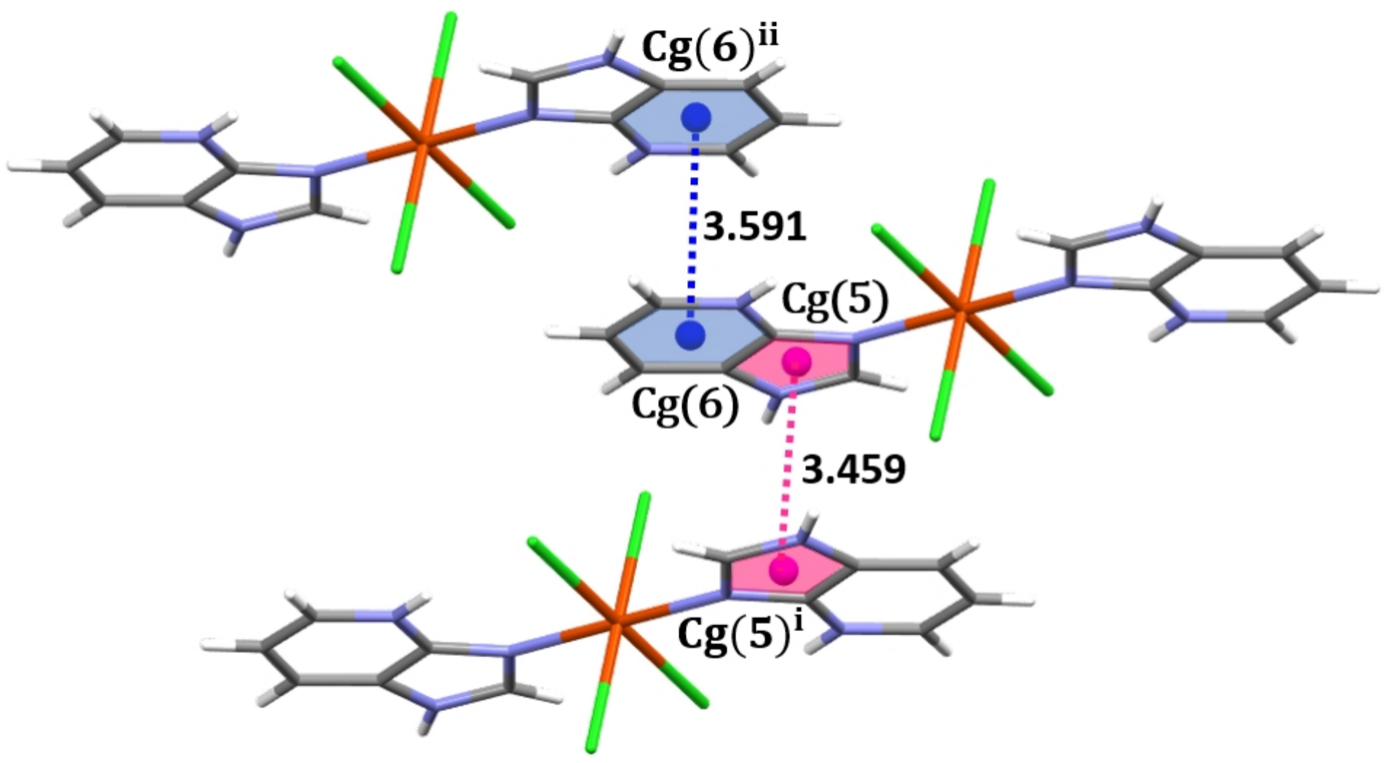
π–π stacking inter­actions in the title complex. *Cg*(5) and *Cg*(6) are the centroids of imidazole rings (pink) and pyridine rings (blue), respectively, [symmetry codes: (i) 1 − *x*, 1 − *y*, 1 − *z*; (ii) 1 − *x*, 1 − *y*, −*z*].

Overall, the crystal packing is reinforced by π–π stacking inter­actions involving both the six-membered pyridine rings (*Cg*6) and the five-membered imidazole rings (*Cg*5) of neighbouring mol­ecules in a parallel-displaced arrangement (Fig. 5 ▸) with centroid–centroid distances of 3.591 (1) and 3.459 (1) Å, respectively

**Figure 6:**
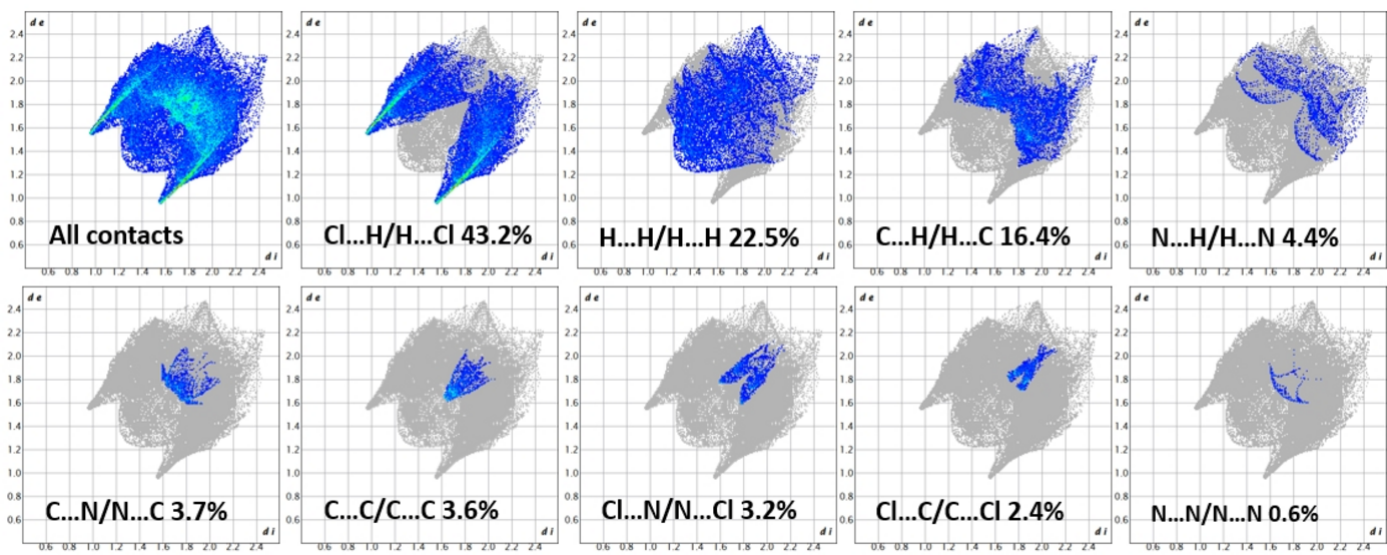
Two-dimensional fingerprint plots for the title compound with the corresponding Hirshfeld surface *d*_norm_ for all contacts and those delineated into specific contacts. The *d*_i_ and *d*_e_ values represent the nearest inter­nal and external distances from specific points on the Hirshfeld surface (in Å).

To gain a deeper insight into the inter­molecular inter­actions responsible for the cohesion and stability of the supra­molecular structure, a Hirshfeld surface (HS) analysis was performed using *Crystal Explorer 21* (Spackman & Jayatilaka, 2009 ▸; Spackman *et al.*, 2021 ▸). The two-dimensional fingerprint plots (Fig. 6 ▸; Spackman & McKinnon, 2002 ▸) show that Cl⋯H/H⋯Cl contacts are the most significant (43.2%), followed by H⋯H (22.5%) and C⋯H/H⋯C (16.4%). Minor contributions arise from H⋯N/N⋯H (4.4%), C⋯N/N⋯C (3.7%), C⋯C (3.6%), and Cl⋯N/N⋯Cl (3.2%), while Cl⋯C/C⋯Cl (2.4%) and N⋯N (0.6%) are negligible. Overall, the HS analysis emphasizes the role of hydrogen bonding and van der Waals inter­actions (Hökelek *et al.*, 2018 ▸; Hathwar *et al.*, 2015 ▸) in consolidating the packing, together with π–π stacking. In addition, it confirms the absence of halogen–halogen contacts, as the Cl⋯Cl contribution is 0% (Moon *et al.*, 2020 ▸).

## 4. Database survey

A Cambridge Structural Database (CSD, version 5.45, November 2023 update; Groom *et al.*, 2016 ▸; Bruno *et al.*, 2002 ▸) search revealed only one reported iron complex with 4-aza­benzimidazole, where the ligand acts in a bridging mode *via* the two imidazole N atoms generating a three-dimensional diamond-like framework (refcode XASGON; Rettig *et al.*, 2000 ▸). A zinc analogue with the same coordination mode has also been described (MIHHOB; Hayashi *et al.*, 2007 ▸).

In contrast, several copper complexes display diverse coordination behaviours. In some cases, the ligand coordinates in a monodentate fashion via the amine nitro­gen [GEZCOF (Domínguez-Martín *et al.*, 2013 ▸); BUNZEQ (Choquesillo-Laza­rte *et al.*, 2010 ▸)], while in others, coordination occurs through the imine nitro­gen, as in the title compound (GEZCIZ; Domínguez-Martín *et al.*, 2013 ▸). Alternatively, 4-aza­benzimidazole can act as a bridging ligand through both the imine and pyridinic nitro­gen atoms, affording dinuclear Cu^II^ paddlewheel-like architectures (TETGIJ and TETGOP; van Albada *et al.*, 2006 ▸).

To date, no structures have been reported with 4-aza­benzimidazole protonated at the pyridinic nitro­gen atom, until the present compound.

## 5. Experimental

All chemicals were commercially available, purchased from Sigma-Aldrich, and used as received without purification.

### 5.1. Synthesis and crystallization

FeCl_2_·4H_2_O (0.198 g, 1 mmol) was dissolved in 10 mL of methanol in the presence of a few drops of ascorbic acid. A solution of 4-aza­benzimidazole (0.238 g, 2 mmol) in 10 mL of aceto­nitrile was then added, resulting in a brown mixture, which was heated under stirring until boiling. The solution was left to evaporate slowly over several days, yielding green crystals of the title compound, suitable for single-crystal X-ray diffraction analysis.

### 5.2. Refinement

Crystal data, data collection and structure refinement details are summarized in Table 3 ▸. All hydrogen atoms were located from difference Fourier maps and refined freely with isotropic displacement parameters.

## Supplementary Material

Crystal structure: contains datablock(s) global, I. DOI: 10.1107/S2056989025010564/vu2014sup1.cif

Structure factors: contains datablock(s) I. DOI: 10.1107/S2056989025010564/vu2014Isup2.hkl

CCDC reference: 2505852

Additional supporting information:  crystallographic information; 3D view; checkCIF report

## Figures and Tables

**Table 1:** Selected geometric parameters (Å, °)

Fe1—Cl1	2.5845 (2)	Fe1—N1	2.2256 (9)
Fe1—Cl2	2.4564 (2)		
			
Cl1—Fe1—Cl2	89.75 (1)	Cl1—Fe1—N1^i^	92.41 (2)
Cl1—Fe1—N1	87.59 (2)	Cl2—Fe1—N1	92.50 (2)
Cl1—Fe1—Cl2^i^	90.25 (1)	Cl2—Fe1—N1^i^	87.50 (2)

Symmetry code: (i) 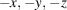.

**Table 2:** Hydrogen-bond geometry (Å, °)

*D*—H⋯*A*	*D*—H	H⋯*A*	*D*⋯*A*	*D*—H⋯*A*
N2—H2⋯Cl2^ii^	0.86 (2)	2.66 (2)	3.3061 (10)	133.0 (17)
N2—H2⋯Cl2^iii^	0.86 (2)	2.56 (2)	3.1862 (9)	130.4 (17)
N3—H3⋯Cl1^i^	0.89 (2)	2.15 (2)	2.9944 (10)	160.7 (19)
C1—H1⋯Cl1	0.942 (15)	2.702 (15)	3.2841 (10)	120.7 (11)
C5—H5⋯Cl2^iv^	0.958 (15)	2.725 (15)	3.6357 (10)	159.1 (12)
C6—H6⋯Cl1^v^	0.954 (15)	2.641 (15)	3.5932 (11)	176.2 (13)

Symmetry codes: (i) 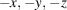; (ii) 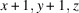; (iii) 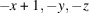; (iv) 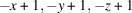; (v) 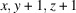.

**Table 3:** Experimental details

Crystal data	
Chemical formula	[FeCl_4_(C_6_H_6_N_3_)_2_]
*M*_r_	437.93
Crystal system, space group	Triclinic, *P* 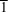
Temperature (K)	100
*a*, *b*, *c* (Å)	6.9242 (2), 7.5223 (2), 8.6704 (2)
α, β, γ (°)	106.967 (2), 98.665 (2), 109.174 (2)
*V* (Å^3^)	392.51 (1)
*Z*	1
Radiation type	Mo *K*α
μ (mm^−1^)	1.65
Crystal size (mm)	0.10 × 0.09 × 0.08

Data collection	
Diffractometer	Nonius KappaCCD
No. of measured, independent and observed [*I* > 2σ(*I*)] reflections	30304, 2818, 2650
*R*_int_	0.026
(sin θ/λ)_max_ (Å^−1^)	0.764

Refinement	
*R*[*F*^2^ > 2σ(*F*^2^)], *wR*(*F*^2^), *S*	0.019, 0.049, 1.07
No. of reflections	2818
No. of parameters	130
H-atom treatment	All H-atom parameters refined
Δρ_max_, Δρ_min_ (e Å^−3^)	0.51, −0.27

Computer programs: *APEX2* and *SAINT* (Bruker, 2024 ▸), *SHELXT* (Sheldrick, 2015*a* ▸), *SHELXL2018/3* (Sheldrick, 2015*b* ▸) and *OLEX2* (Dolomanov *et al.*, 2009 ▸).

## supplementary crystallographic information

### Tetrachloridobis(1H-imidazo[4,5-b]pyridin-4-ium-κN3)iron(II) Crystal data

**Table:**

[FeCl_4_(C_6_H_6_N_3_)_2_]	*Z* = 1
*M**_r_* = 437.93	*F*(000) = 220
Triclinic, *P*1	*D*_x_ = 1.853 Mg m^−^^3^
Hall symbol: -P 1	Mo *K*α radiation, λ = 0.71073 Å
*a* = 6.9242 (2) Å	Cell parameters from 30213 reflections
*b* = 7.5223 (2) Å	θ = 3.1–32.9°
*c* = 8.6704 (2) Å	µ = 1.65 mm^−^^1^
α = 106.967 (2)°	*T* = 100 K
β = 98.665 (2)°	Prism, green
γ = 109.174 (2)°	0.1 × 0.09 × 0.08 mm
*V* = 392.51 (1) Å^3^	

### Tetrachloridobis(1H-imidazo[4,5-b]pyridin-4-ium-κN3)iron(II) Data collection

**Table:**

Nonius KappaCCD diffractometer	*R*_int_ = 0.026
Radiation source: fine-focus sealed tube	θ_max_ = 32.9°, θ_min_ = 3.1°
ω scans	*h* = −10→10
30304 measured reflections	*k* = −11→11
2818 independent reflections	*l* = −12→13
2650 reflections with *I* > 2σ(*I*)	

### Tetrachloridobis(1H-imidazo[4,5-b]pyridin-4-ium-κN3)iron(II) Refinement

**Table:**

Refinement on *F*^2^	0 restraints
Least-squares matrix: full	Hydrogen site location: difference Fourier map
*R*[*F*^2^ > 2σ(*F*^2^)] = 0.019	All H-atom parameters refined
*wR*(*F*^2^) = 0.049	*w* = 1/[σ^2^(*F*_o_^2^) + (0.0225*P*)^2^ + 0.1909*P*] where *P* = (*F*_o_^2^ + 2*F*_c_^2^)/3
*S* = 1.07	(Δ/σ)_max_ = 0.001
2818 reflections	Δρ_max_ = 0.51 e Å^−^^3^
130 parameters	Δρ_min_ = −0.26 e Å^−^^3^

### Tetrachloridobis(1H-imidazo[4,5-b]pyridin-4-ium-κN3)iron(II) Special details

**Table:**

Geometry. Bond distances, angles etc. have been calculated using the rounded fractional coordinates. All su's are estimated from the variances of the (full) variance-covariance matrix. The cell esds are taken into account in the estimation of distances, angles and torsion angles

### Tetrachloridobis(1H-imidazo[4,5-b]pyridin-4-ium-κN3)iron(II) Fractional atomic coordinates and isotropic or equivalent isotropic displacement parameters (Å^2^)

**Table:**

	*x*	*y*	*z*	*U*_iso_*/*U*_eq_	
Fe1	0.00000	0.00000	0.00000	0.0091 (1)	
Cl1	0.11270 (3)	−0.15421 (3)	−0.26126 (3)	0.0118 (1)	
Cl2	0.10569 (3)	−0.21585 (3)	0.12967 (3)	0.0112 (1)	
N1	0.32581 (13)	0.24252 (12)	0.09987 (10)	0.0104 (2)	
N2	0.65992 (13)	0.42459 (12)	0.10840 (10)	0.0110 (2)	
N3	0.30049 (13)	0.49999 (12)	0.33328 (10)	0.0116 (2)	
C1	0.48880 (15)	0.24861 (14)	0.03344 (12)	0.0112 (2)	
C2	0.40177 (14)	0.42778 (13)	0.22624 (11)	0.0096 (2)	
C3	0.61149 (14)	0.54528 (14)	0.23671 (11)	0.0101 (2)	
C4	0.72138 (15)	0.73656 (14)	0.35954 (12)	0.0127 (2)	
C5	0.61028 (16)	0.80452 (15)	0.46871 (12)	0.0142 (2)	
C6	0.40167 (16)	0.68616 (15)	0.45319 (12)	0.0136 (2)	
H1	0.486 (2)	0.141 (2)	−0.0572 (18)	0.014 (3)*	
H2	0.779 (3)	0.447 (3)	0.084 (2)	0.025 (4)*	
H3	0.169 (3)	0.421 (3)	0.324 (2)	0.028 (4)*	
H4	0.867 (2)	0.817 (2)	0.3706 (19)	0.018 (4)*	
H5	0.676 (2)	0.934 (2)	0.5580 (19)	0.019 (4)*	
H6	0.320 (2)	0.728 (2)	0.5245 (19)	0.018 (3)*	

### Tetrachloridobis(1H-imidazo[4,5-b]pyridin-4-ium-κN3)iron(II) Atomic displacement parameters (Å^2^)

**Table:**

	*U*^11^	*U*^22^	*U*^33^	*U*^12^	*U*^13^	*U*^23^
Fe1	0.0081 (1)	0.0083 (1)	0.0104 (1)	0.0026 (1)	0.0036 (1)	0.0031 (1)
Cl1	0.0110 (1)	0.0115 (1)	0.0125 (1)	0.0038 (1)	0.0055 (1)	0.0035 (1)
Cl2	0.0110 (1)	0.0109 (1)	0.0127 (1)	0.0049 (1)	0.0048 (1)	0.0043 (1)
N1	0.0098 (3)	0.0095 (3)	0.0113 (3)	0.0034 (3)	0.0035 (3)	0.0030 (3)
N2	0.0089 (3)	0.0120 (3)	0.0127 (3)	0.0042 (3)	0.0048 (3)	0.0044 (3)
N3	0.0112 (3)	0.0114 (3)	0.0131 (3)	0.0043 (3)	0.0058 (3)	0.0047 (3)
C1	0.0105 (4)	0.0112 (4)	0.0121 (4)	0.0045 (3)	0.0037 (3)	0.0038 (3)
C2	0.0092 (4)	0.0092 (4)	0.0108 (3)	0.0033 (3)	0.0034 (3)	0.0042 (3)
C3	0.0095 (4)	0.0109 (4)	0.0106 (4)	0.0040 (3)	0.0033 (3)	0.0047 (3)
C4	0.0117 (4)	0.0112 (4)	0.0131 (4)	0.0022 (3)	0.0023 (3)	0.0046 (3)
C5	0.0161 (4)	0.0109 (4)	0.0133 (4)	0.0041 (3)	0.0033 (3)	0.0030 (3)
C6	0.0166 (4)	0.0125 (4)	0.0127 (4)	0.0069 (3)	0.0058 (3)	0.0036 (3)

### Tetrachloridobis(1H-imidazo[4,5-b]pyridin-4-ium-κN3)iron(II) Geometric parameters (Å, º)

**Table:**

Fe1—Cl1	2.5845 (2)	N3—C6	1.3488 (14)
Fe1—Cl2	2.4564 (2)	C2—C3	1.4030 (15)
Fe1—N1	2.2256 (9)	N2—H2	0.86 (2)
Fe1—Cl1^i^	2.5845 (2)	N3—H3	0.89 (2)
Fe1—Cl2^i^	2.4564 (2)	C3—C4	1.3877 (14)
Fe1—N1^i^	2.2256 (9)	C4—C5	1.3944 (15)
N1—C1	1.3360 (14)	C5—C6	1.3871 (16)
N1—C2	1.3686 (13)	C1—H1	0.942 (15)
N2—C1	1.3469 (14)	C4—H4	0.964 (15)
N2—C3	1.3774 (13)	C5—H5	0.958 (15)
N3—C2	1.3418 (13)	C6—H6	0.954 (15)
			
Cl1—Fe1—Cl2	89.75 (1)	N1—C2—N3	127.71 (9)
Cl1—Fe1—N1	87.59 (2)	N1—C2—C3	111.74 (9)
Cl1—Fe1—Cl1^i^	180.00	N3—C2—C3	120.54 (9)
Cl1—Fe1—Cl2^i^	90.25 (1)	C3—N2—H2	128.4 (13)
Cl1—Fe1—N1^i^	92.41 (2)	C1—N2—H2	124.0 (13)
Cl2—Fe1—N1	92.50 (2)	N2—C3—C4	134.70 (10)
Cl1^i^—Fe1—Cl2	90.25 (1)	C2—C3—C4	121.25 (9)
Cl2—Fe1—Cl2^i^	180.00	N2—C3—C2	104.04 (8)
Cl2—Fe1—N1^i^	87.50 (2)	C2—N3—H3	118.1 (12)
Cl1^i^—Fe1—N1	92.41 (2)	C6—N3—H3	122.1 (12)
Cl2^i^—Fe1—N1	87.50 (2)	C3—C4—C5	116.15 (10)
N1—Fe1—N1^i^	180.00	C4—C5—C6	121.22 (10)
Cl1^i^—Fe1—Cl2^i^	89.75 (1)	N3—C6—C5	121.01 (10)
Cl1^i^—Fe1—N1^i^	87.59 (2)	N1—C1—H1	123.8 (9)
Cl2^i^—Fe1—N1^i^	92.50 (2)	N2—C1—H1	122.6 (9)
Fe1—N1—C1	126.40 (7)	C3—C4—H4	122.1 (9)
Fe1—N1—C2	129.89 (7)	C5—C4—H4	121.7 (9)
C1—N1—C2	103.25 (9)	C4—C5—H5	121.1 (9)
C1—N2—C3	107.32 (9)	C6—C5—H5	117.6 (9)
C2—N3—C6	119.81 (9)	N3—C6—H6	115.3 (9)
N1—C1—N2	113.63 (9)	C5—C6—H6	123.7 (9)
			
Cl1—Fe1—N1—C1	−7.58 (8)	C3—N2—C1—N1	1.16 (12)
Cl1—Fe1—N1—C2	163.34 (8)	C1—N2—C3—C2	−1.28 (11)
Cl2—Fe1—N1—C1	82.07 (8)	C1—N2—C3—C4	177.73 (11)
Cl2—Fe1—N1—C2	−107.02 (8)	C6—N3—C2—N1	178.75 (10)
Cl1^i^—Fe1—N1—C1	172.42 (8)	C6—N3—C2—C3	−0.74 (14)
Cl1^i^—Fe1—N1—C2	−16.66 (8)	C2—N3—C6—C5	−0.45 (15)
Cl2^i^—Fe1—N1—C1	−97.93 (8)	N1—C2—C3—N2	1.06 (11)
Cl2^i^—Fe1—N1—C2	72.98 (8)	N1—C2—C3—C4	−178.12 (9)
Fe1—N1—C1—N2	172.38 (7)	N3—C2—C3—N2	−179.38 (9)
C2—N1—C1—N2	−0.47 (11)	N3—C2—C3—C4	1.45 (15)
Fe1—N1—C2—N3	7.58 (15)	N2—C3—C4—C5	−179.78 (11)
Fe1—N1—C2—C3	−172.89 (7)	C2—C3—C4—C5	−0.90 (15)
C1—N1—C2—N3	−179.92 (10)	C3—C4—C5—C6	−0.28 (15)
C1—N1—C2—C3	−0.39 (11)	C4—C5—C6—N3	0.98 (16)

Symmetry code: (i) −*x*, −*y*, −*z*.

### Tetrachloridobis(1H-imidazo[4,5-b]pyridin-4-ium-κN3)iron(II) Hydrogen-bond geometry (Å, º)

**Table:**

*D*—H···*A*	*D*—H	H···*A*	*D*···*A*	*D*—H···*A*
N2—H2···Cl2^ii^	0.86 (2)	2.66 (2)	3.3061 (10)	133.0 (17)
N2—H2···Cl2^iii^	0.86 (2)	2.56 (2)	3.1862 (9)	130.4 (17)
N3—H3···Cl1^i^	0.89 (2)	2.15 (2)	2.9944 (10)	160.7 (19)
C1—H1···Cl1	0.942 (15)	2.702 (15)	3.2841 (10)	120.7 (11)
C5—H5···Cl2^iv^	0.958 (15)	2.725 (15)	3.6357 (10)	159.1 (12)
C6—H6···Cl1^v^	0.954 (15)	2.641 (15)	3.5932 (11)	176.2 (13)

Symmetry codes: (i) −*x*, −*y*, −*z*; (ii) *x*+1, *y*+1, *z*; (iii) −*x*+1, −*y*, −*z*; (iv) −*x*+1, −*y*+1, −*z*+1; (v) *x*, *y*+1, *z*+1.

### Geometric parameters of π··· π stacking (Å,°)

**Table:**

Cg ⋯ Cg (Ring)	Cg ⋯ Cg (Å)	α(°)	β(°)	γ(°)
Cg(5) ⋯ Cg(5)i	3.459 (1)	0.00	23.13	23.13
Cg(6) ⋯ Cg(6)ii	3.591 (1)	0.00	21.67	21.67

Symmetry codes: (i) 1-x,1-y,-z, (ii) 1-x,1-y,1-z Cg(5): center of gravity of imidazole ring. Cg(6): center of gravity of pyridine ring. α: dihedral angle between ring planes. β, γ: Slipping angles defined by centroid–centroid vector and the normal to the plane of the ring.
